# Bony metastases from breast cancer - a study of foetal antigen 2 as a blood tumour marker

**DOI:** 10.1186/1477-7819-8-38

**Published:** 2010-05-13

**Authors:** Kwok-Leung Cheung, Ray K Iles, John FR Robertson

**Affiliations:** 1 Division of Breast Surgery, University of Nottingham, Nottingham, UK; 2 Williamson Laboratory, St Bartholomew's Hospital, London, UK

## Abstract

**Background:**

Foetal antigen 2 (FA-2), first isolated in the amniotic fluid, was shown to be the circulating form of the aminopropeptide of the alpha 1 chain of procollagen type I. Serum concentrations of FA-2 appeared to be elevated in a number of disorders of bone metabolism. This paper is the first report of its role as a marker of bone metabolism in metastatic breast cancer.

**Methods:**

Serum FA-2 concentrations were measured by radioimmunoassay in 153 women with different stages of breast cancer and in 34 normal controls.

**Results:**

Serum FA-2 was significantly elevated in women with bony metastases (*p *< 0.015). Its levels were not significantly different among women with non-bony metastases, with non-metastatic disease, as well as among normal controls.

**Conclusions:**

FA-2 is a promising blood marker of bone metabolism. Further studies to delineate its role in the diagnosis and management of bony metastases from breast cancer are required.

## Background

Foetal antigen 2 (FA-2) was first isolated in the amniotic fluid [1] and subsequent structural characterisation showed that it was the circulating form of the aminopropeptide of the alpha 1 chain of procollagen type I [2]. As will be discussed later, serum concentrations of FA-2 appeared to be elevated in a number of disorders with altered bone metabolism. The bone is the commonest site of involvement in metastatic breast cancer. Bone metabolism, including formation and resorption, is influenced by the disease process, by anti-cancer therapies (cytotoxic or endocrine therapy) and by the recently popularised use of bisphosphonates. A circulating marker which can better reflect the process of bone metabolism is needed in the management of women with bony metastases from breast cancer, in addition to currently available markers of tumour load (*eg *MUC1 mucin measured as cancer antigen 15.3 (CA15.3), carcinoembryonic antigen (CEA)). FA-2 seems to be a promising marker of such kind. This paper analyses the serum levels of FA-2 in different stages of breast cancer and examines its role as a marker of bone metabolism in metastatic breast cancer.

## Methods

### Patients

Blood samples were obtained with informed consents from the following four groups of women seen in the Nottingham Breast Unit:

1. Normal - This included women from two sources. The first group were women attending the Screening Assessment Clinic according to the UK National Health Service Breast Screening Programme and were proven after assessment (clinical, radiological and/or histological) to have no malignancy in the breast. The second group of women attended the Benign Lumps Clinic and they had been proven by investigations (including imaging and histology) to have either benign lump(s) or no abnormality in the breast.

2. Women with primary breast cancer (PBC) - All had tumour < 5 cm and blood samples were taken at the preoperative assessment clinic prior to surgery.

3. Women with locally advanced primary breast cancer (LAPC) - These women had LAPC as defined by having tumour > 5 cm and/or other features of locally advanced disease (*eg *inflammatory cancer, fixation to chest wall, ulcerating tumour) without any evidence of distant metastases and attended the LAPC Clinic. Blood samples were taken when the tumour was still *in situ*.

4. Women with advanced breast cancer (ABC) - These were women attending the ABC Clinic and all had distant metastases.

### Preparation of Serum Samples

Blood obtained by venesection was collected in plain tubes, allowed to stand for at least 30 minutes and then centrifuged at 2,500 revolutions per minute for 20 minutes. Serum was pipetted into 1-ml aliquots and stored in the freezer at -20°C.

### FA-2 Assays

The serum samples were transported at -20°C to the Williamson Laboratory at St Bartholomew's Hospital. FA-2 radioimmunoassays were carried out as previously described [3]. The assays were performed in a blind manner with aliquots tagged with a sample number without any clinical information.

### Statistical Methods

Statistical analysis was carried out using the standardised biomedical computer programme SPSS for Windows (SPSS UK Ltd). The ANOVA test was used for multiple group comparison of the mean values. Statistically significant difference was defined by *p *< 0.05.

The authors confirm that approval has been obtained from Local Research Ethics Committee to conduct this study on blood markers in breast cancer.

## Results

**Figure 1:**
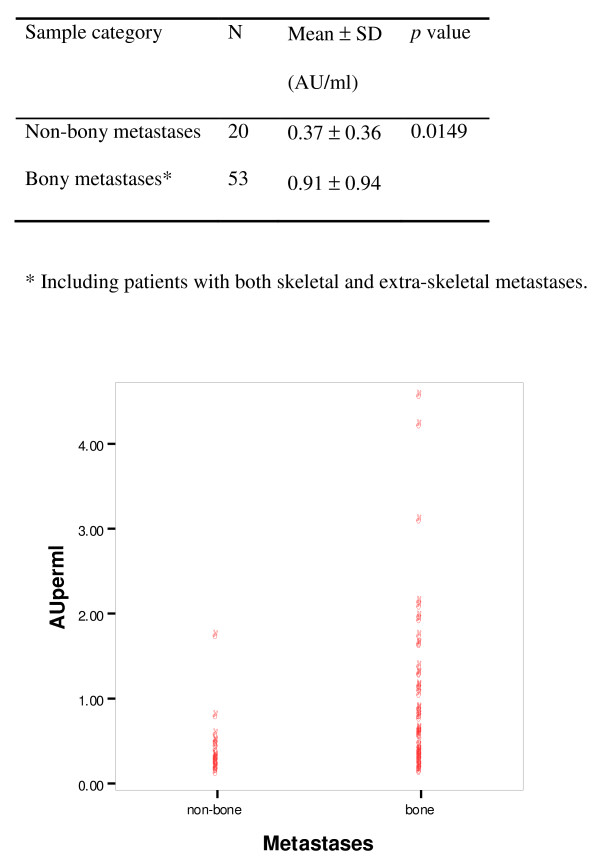
**Comparison of FA-2 levels between bone and non-bone metastases**.

The mean values of serum FA-2 levels in all four groups of women were summarised in Table 1. There was no difference in FA-2 levels among normal women and women with breast cancer which was still confined to the breast (*ie *PBC and LAPC) (Tables 1 and 2). Nevertheless, when all stages of cancer were taken into consideration, FA-2 levels appeared to be significantly elevated in cancer patients when compared to normal women and this was due to marked elevation in women with metastatic breast cancer (Table 2). Women with metastatic disease had a much higher value of FA-2 than those without (Table 3) and this was due to the significant elevation of FA-2 in women with bony metastases (Figure 1).

**Table 1:** Mean Values of FA-2 for All Women

Sample category	N	Mean ± SD (AU/ml)
Normal	34	0.21 ± 0.09
PBC	35	0.18 ± 0.08
LAPC	38	0.33 ± 0.67
ABC	80	0.85 ± 1.34
All	187	

**Table 2:** Comparison of FA-2 Levels between Different Groups

Sample category	*p *value
PBC	0.19
LAPC	

	

Sample category	*p *value

PBC	0.0037
ABC	

	

Sample category	*p *value

LAPC	0.0243
ABC	

**Table 3:** Comparison of FA-2 Levels between Metastatic and Non-Metastatic Cancers

Sample category	N	Mean ± SD (AU/ml)	*p *value
Non-metastatic	73	0.26 ± 0.49	0.0004
Metastatic	80	0.85 ± 1.34	

In conclusion, the results suggested that FA-2 was significantly elevated only in the subgroup of women with bony metastases.

## Discussion

Blood tumour markers in breast cancer have been known for decades. In contrast to markers in the primary tumour tissue, blood tumour markers reflect a dynamic situation and their measurements can be repeated as required. The use of blood tumour markers is most established in the diagnosis and monitoring of symptomatic metastatic disease. In the diagnosis of metastatic breast cancer, CA15.3 assay has been shown to be superior with CEA being the next most clinically useful marker [4]. The sensitivity can be further increased when a panel of three markers *ie *CA15.3, CEA and ESR are used [5-8]. While the usefulness of blood tumour markers is well established in advanced breast cancer, active research, both clinical and laboratory, is ongoing to refine the measurements of existing markers, to explore newer markers and to develop better marker assays, aiming to optimise their use in advanced disease as well as to exploit their use in screening and diagnosis of early primary breast cancer.

Markers of bone metabolism are among the new markers which are being investigated. Traditional markers of bone metabolism include serum alkaline phosphatase, serum and urinary calcium, urinary hydroxproline *etc*. Markers of collagen synthesis have been evaluated as bone markers for metastatic bone disease due to breast cancer. The most abundant protein in bone is type I collagen. During its formation two extension peptides from the procollagen molecule, carboxy- and aminoterminal propeptides (PICP and PINP) are released into the circulation and they are markers of bone formation. Type I collagen carboxyterminal telopeptide (ICTP) is formed during bone collagen breakdown and is again liberated into the circulation. Its level in the serum therefore reflects bone resorption. ICTP has a high specificity though relatively low sensitivity and is the best bone metabolism marker evaluated [9,10]. Further studies to evaluate the cost-effectiveness of measuring these markers and to explore newer markers of bone metabolism are required [11].

After its isolation from the amniotic fluid, FA-2 was found elevated in serum of patients with renal osteodystrophy [12] and with primary hyperparathyroidism [13]. Patients with the latter had FA-2 levels dropped significantly after surgical removal of the parathyroid glands [13]. All these have suggested FA-2 as a possible marker to evaluate bone metabolism. Evidence of FA-2 synthesis by foetal osteoblasts shown using immunohistochemical staining techniques has substantiated this potential role [14].

The present study is the first report of the measurement of serum FA-2 in different stages of breast cancer. It showed that serum FA-2 was elevated distinctly in women with bony metastases. Its levels were significantly lower in women without metastases including normal controls. The fact that the mean value in women with metastases was significantly higher that in women without could entirely be explained by the inclusion of women with bony metastases in the former group. The mean value in women with non-bony metastases was virtually similar to that of those without metastases. In essence serum FA-2 has been found to be significantly elevated only in the subgroup of women with bony metastases. These preliminary data point out that FA-2 is a potential helpful blood marker for bony metastases from breast cancer. It would therefore appear that serum FA-2 measurement may be useful in the diagnosis of bony metastases. Whether it will be shown to be superior to existing markers and/or radiological methods remains to be elucidated.

The other role of tumour marker measurement is in the monitoring of therapy. In the present era when the use of bisphosphonates has been popularised in the management of bone metastases for breast cancer, markers of bone metabolism might provide a measurement of the effect of sclerosis on the bone while conventional blood markers such as CA15.3 and CEA reflect the efficacy of anti-cancer therapy on tumour mass. In these ways new markers have a complementary rather than an exclusive role in the diagnosis and monitoring of breast cancer [11].

Given the preliminary results from this observational study which has its statistical limitations due to its small size, further studies are therefore required to define in details the exact value of serum FA-2 measurement in bony metastases from breast cancer. Comparison with conventional markers of tumour mass (*eg *CA15.3, CEA) and known novel markers of bone metabolism (*eg *PICP, PINP, ICTP) (both in the diagnosis and in the monitoring of response to systemic therapy), and identification of the pattern of changes of serum FA-2 levels in relation to bisphosphonate therapy and events such as hypercalcaemia are areas that need to be explored before the use of FA-2 could be incorporated into daily clinical practice.

## Abbreviations

FA-2: Foetal antigen 2; CA15.3: Cancer antigen 15.3; CEA: Carcinoembryonic antigen; PBC: Primary breast cancer; LAPC: Locally advanced primary breast cancer; ABC: Advanced breast cancer; PICP: Carboxyterminal propeptide of type I procollagen; PINP: Aminoterminal propeptide of type I procollagen; ICTP: Type I collagen carboxyterminal telo peptide.

## Competing interests

The authors declare that they have no competing interests.

## Authors' contributions

KLC performed the statistical analysis and drafted the manuscript. All patients were under the care of KLC and JFRR who were responsible for collecting blood samples and clinical data. RKI was responsible for carrying out the assay for FA-2. JFRR conceived of the study. All participated in the design; read and approved the final manuscript.
